# Supplementary material for: Identifying tumor type and cell type-specific gene expression alterations in pediatric central nervous system tumors
Source: Nat Commun. 2024 Apr 30;15:3634. doi: 10.1038/s41467-024-47712-8 (PMC11061189; doi:10.1038/s41467-024-47712-8)
Supplement: Supplementary file 1 — Supplementary Information [file 41467_2024_47712_MOESM1_ESM.pdf]

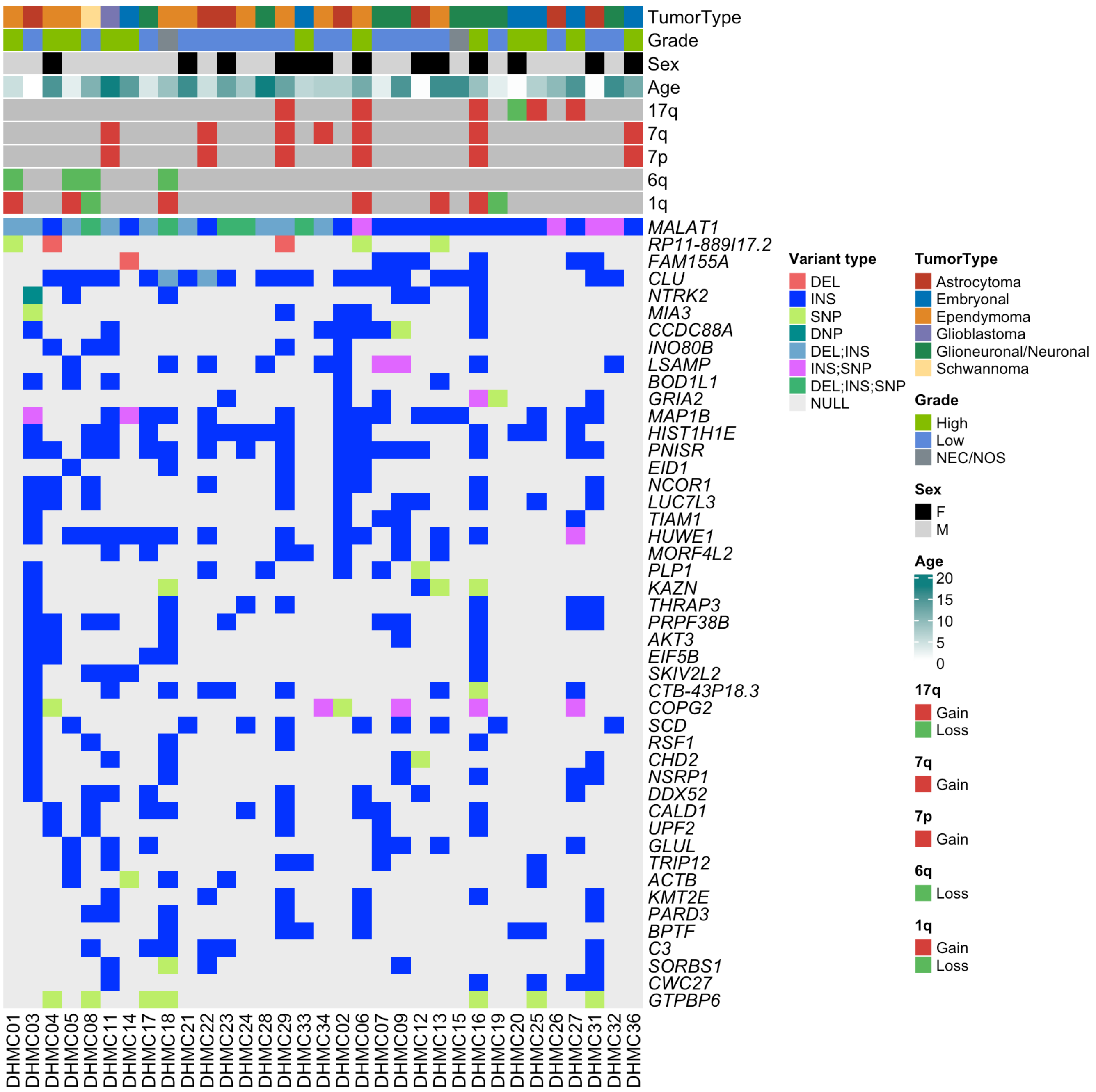

Supplementary Figure 1. Genetic and cytogenic characteristics of pediatric CNS tumors. Heatmap of presence of somatic genetic variant in genes with most variants in the cohort. Cells are colored by the somatic variant type (i.e. deletion, insertion, SNP, etc). Light gray NULL cells indicate no variant present. Horizontal tracking bars correspond to each patient's age, gender, grade, tumor type, and copy number variations in select chromosomes. Genetic variants were determined by bulk RNA-seq. Copy number variations were determined by inference from DNA methylation arrays. **DEL:** deletion; **INS:** insertion; **SNP**: single nucleotide polymorphism; **DBP:** double nucleotide polymorphism.

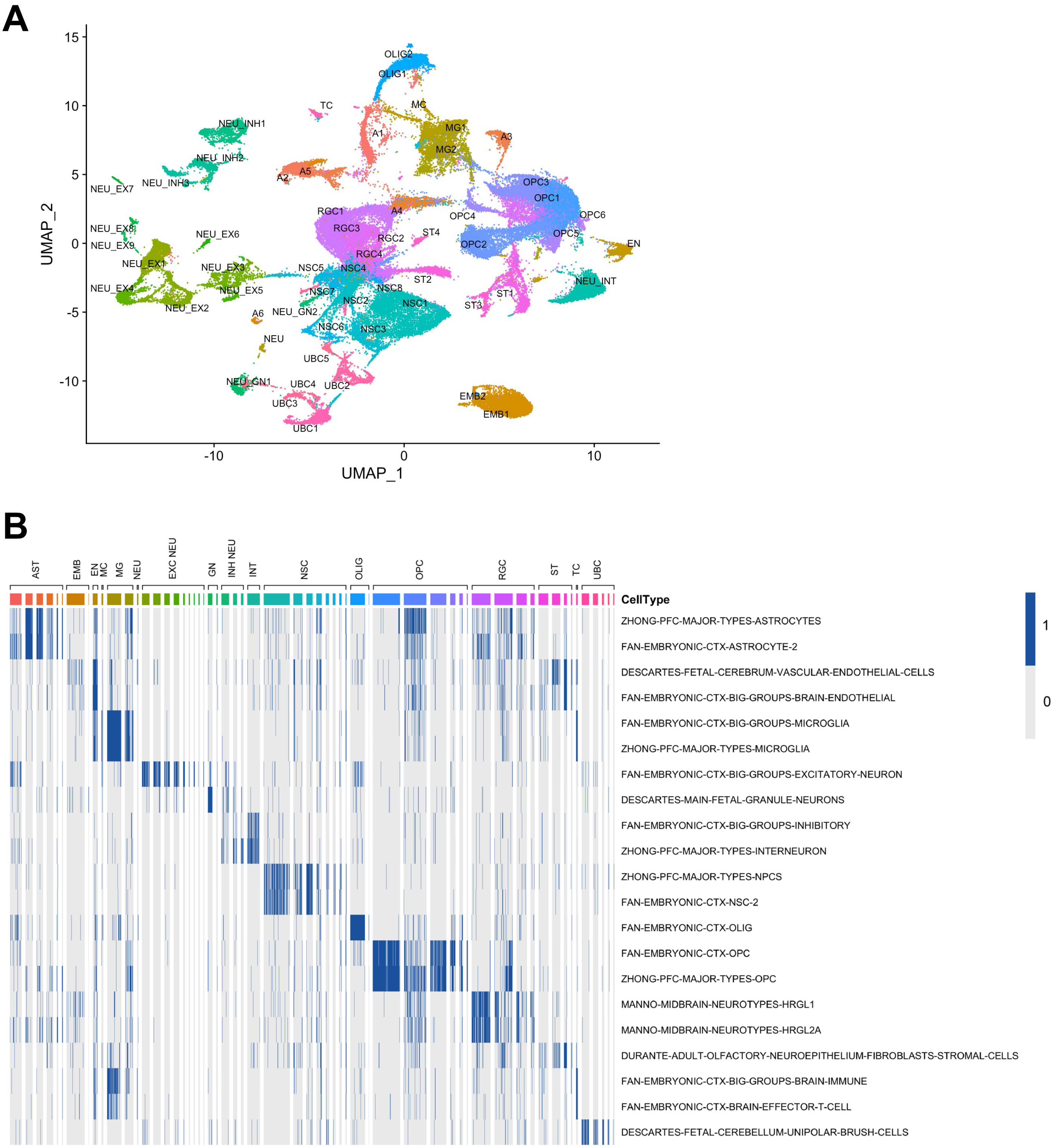

Supplementary Figure 2. Cell type classification of pediatric CNS nuclei. A) UMAP visualization of the 58 clusters identified through Seurat *FindClusters*. **B)** Heatmap of enrichment of cell type specific pathways for each nuclei, determined by Variance-adjusted Mahalanobis enrichment method. 0 indicates pathway is not statistically significantly enriched. 1 indicates pathway is statistically significantly enriched. Tracking bar indicates cell cluster identity from the UMAP in **1A**.

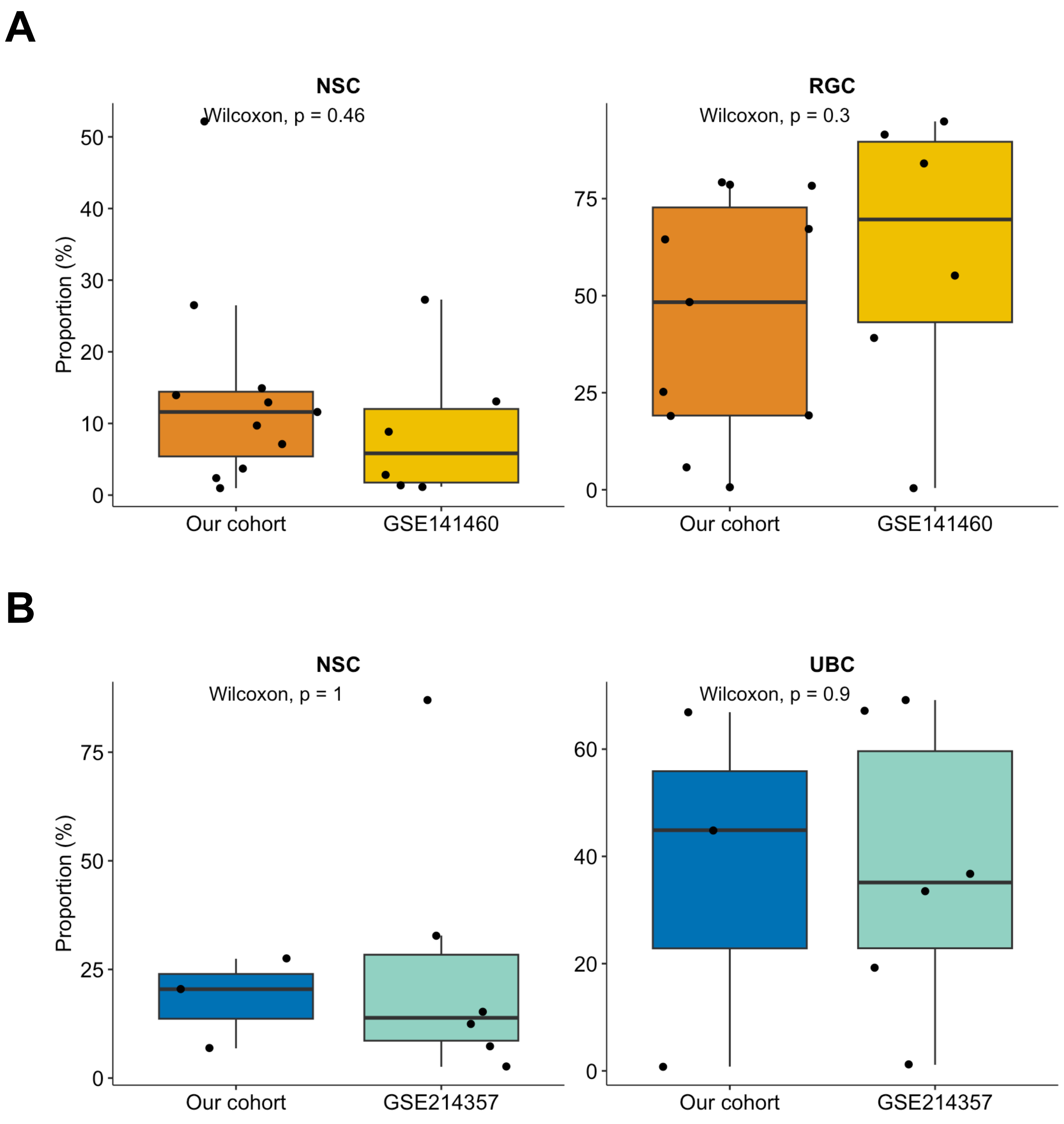

Supplementary Figure 3. Comparison of cell proportions with independent cohorts. **A)** Cell proportions of NSC and RGC in the ependymoma of our cohort and in GSE141460. **B)** Cell proportions of NSC and UBC in the medulloblastoma of our cohort and in GSE214357. Cell type proportion comparisons between our cohort and publicly available datasets were made with Wilcoxon signed rank test. In the boxplot, the low ends of the segment indicate the minimum and the high ends of the segment indicate the maximum. Lower bounds of the box indicate the 25th percentile and the higher bounds of the box indicate the 75th percentile. Segment in the middle is the median.

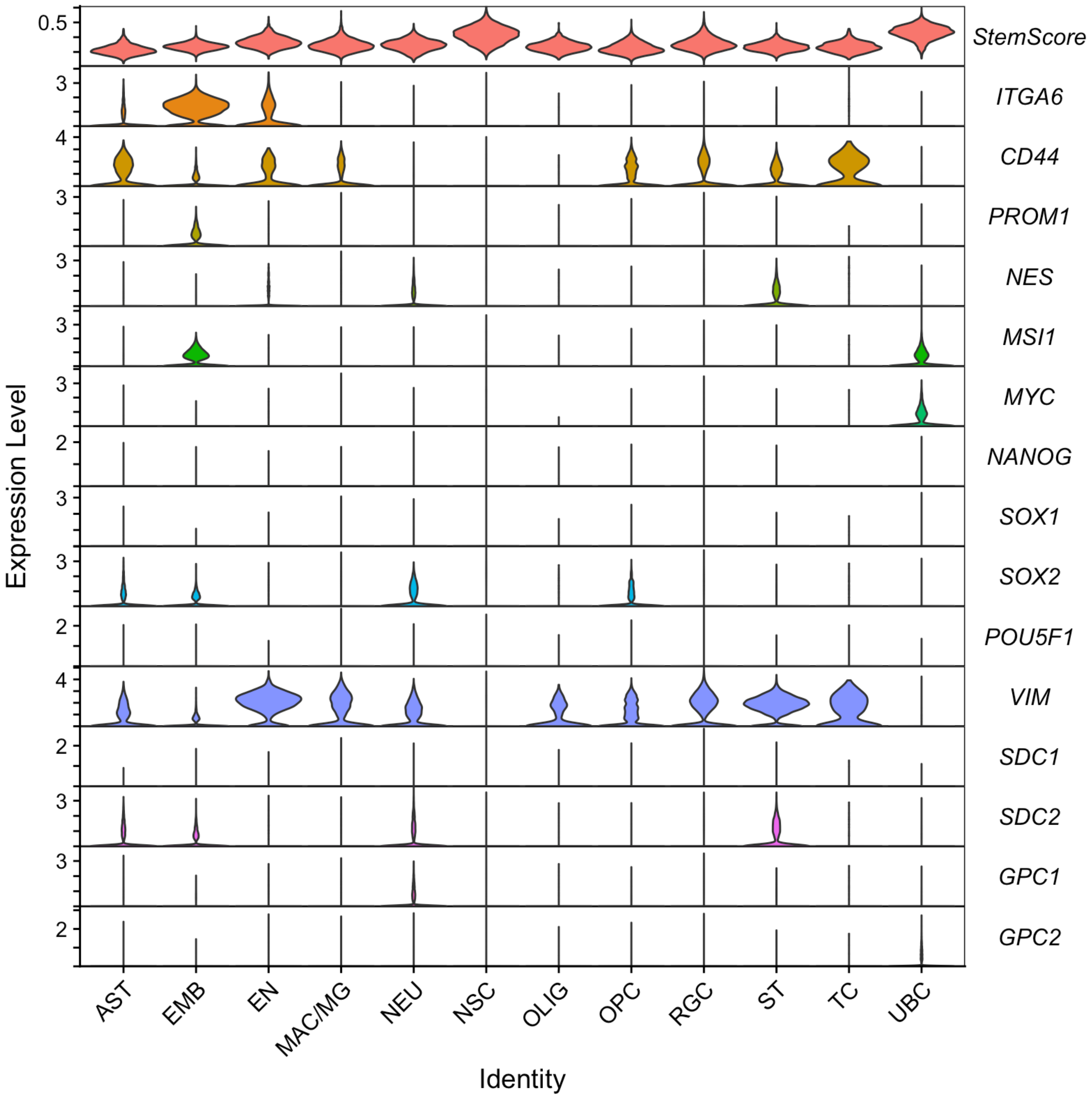

Supplementary Figure 4. Stemness related gene expression levels for each cell type. Violin plots of expression levels of commonly used markers to isolate cancer stem cells and stemness score calculated from set of stem cell associated genes identified in Tirosh et al for each major cell type[35].

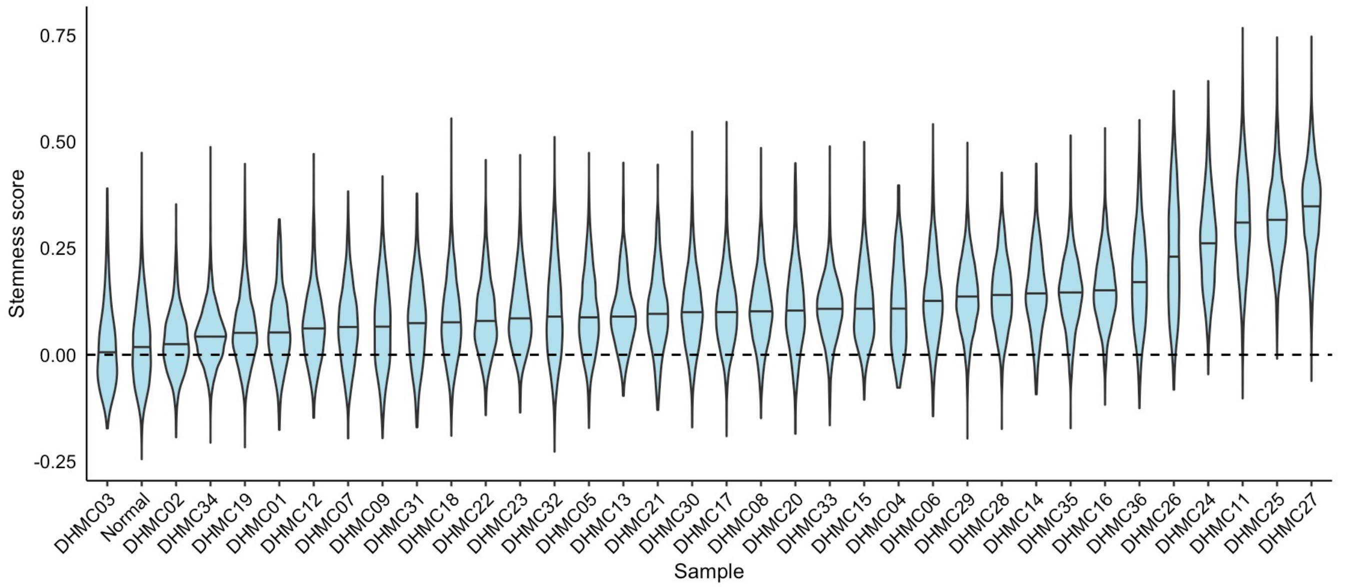

Supplementary Figure 5. Violin plots of the stemness scores of all nuclei for each sample.

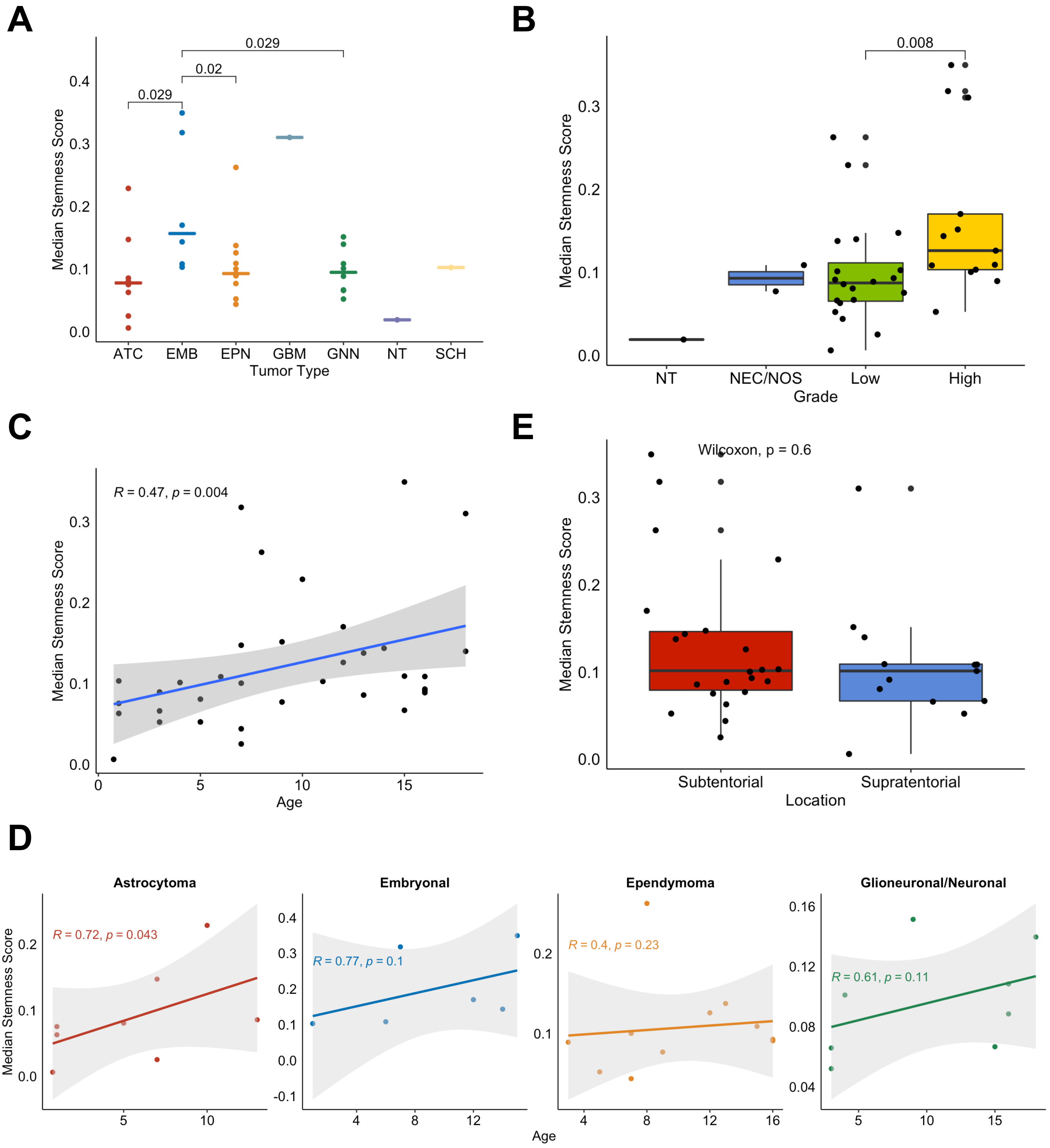

Supplementary Figure 6. Stemness scores by clinical variables. A) Median stemness score distribution by tumor types. Horizontal line for each tumor type indicates median stemness score per tumor type. Comparisons between embryonal tumors and other tumor types were tested with Wilcoxon rank-sum test. **B)** Median stemness score distribution by grade. Comparison between median stemness scores of low and high grade conducted using Wilcoxon rank-sum test. **C)** Correlation between age at diagnosis and median stemness score of tumors. Correlation calculated using the Spearman rank method. Linear regression line and 95% confidence interval indicated by the blue line and gray band, respectively. **D)** Correlation between age and median stemness score separated by each tumor type. Correlation calculated using the Spearman rank method. Linear regression line and 95% confidence interval indicated by the blue line and gray band, respectively. **E)** Median stemness score distribution by tumor location class. Comparison between median stemness scores of subtentorial and supratentorial regions conducted using Wilcoxon rank-sum test. In the boxplots, the low ends of the segment indicate the minimum and the high ends of the segment indicate the maximum. Lower bounds of the box indicate the 25th percentile and the higher bounds of the box indicate the 75th percentile. Segment in the middle is the median.

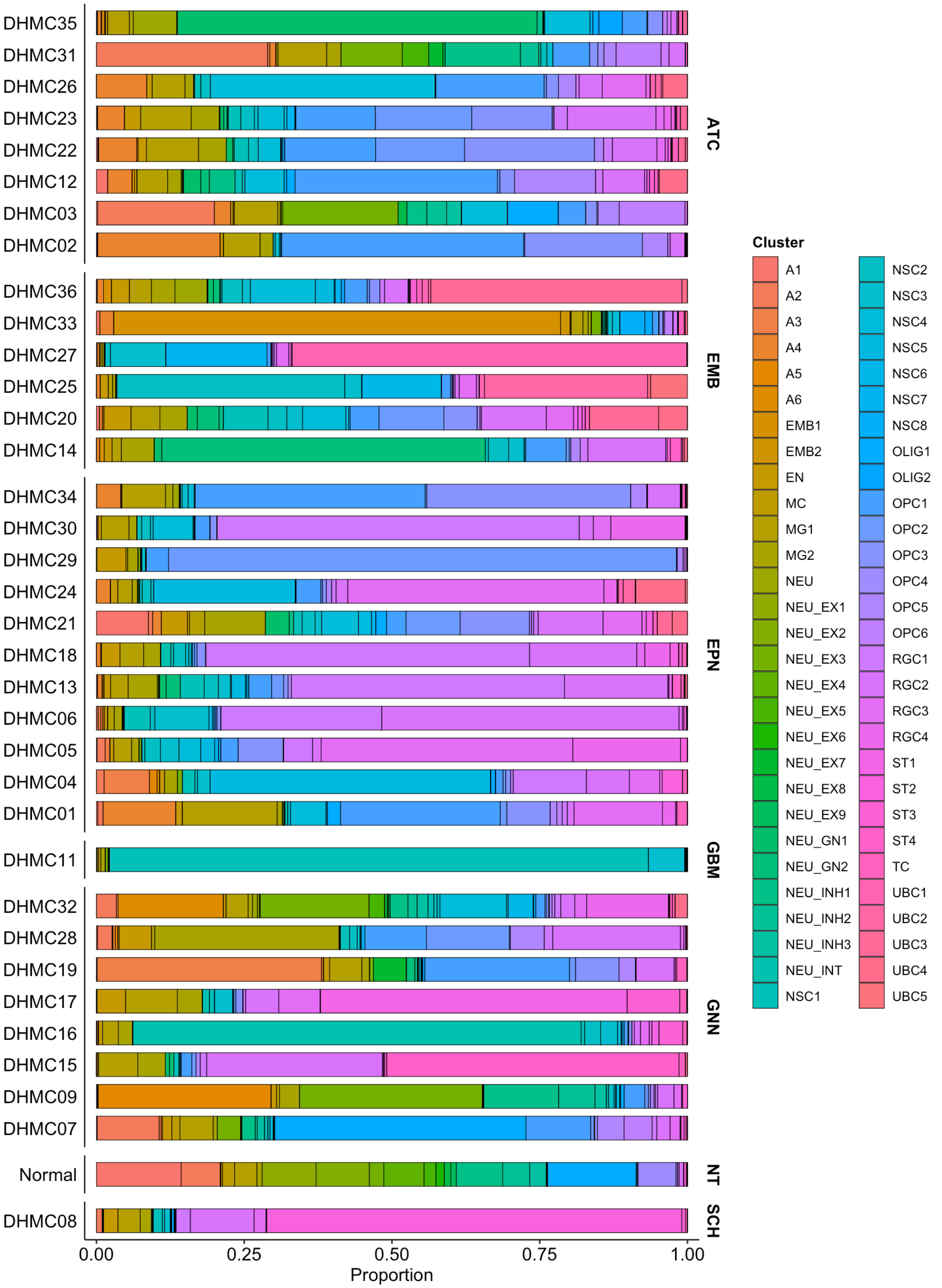

Supplementary Figure 7. Distribution of cell types present per sample, categorized by tumor types. A: Astrocyte; **EMB**: Embryonal tumor cells; **EN**: Endothelial cells; **MC:** Macrophage; **MG**: Microglia; **NEU**: Neuron; **NEU_EX**: Excitatory neuron; **NEU_GN**: Granular neuron; **NEU_INH**: Inhibitory neuron; **NEU_INT**: Interneuron; **NSC**: Neural stem cell; **OLIG**: Oligodendrocyte; **OPC**: Oligodendrocyte precursor cell; **RGC**: Radial glial cell; **ST**: Stromal cell; **TC**: T cell; **UBC**: Unipolar brush cell.

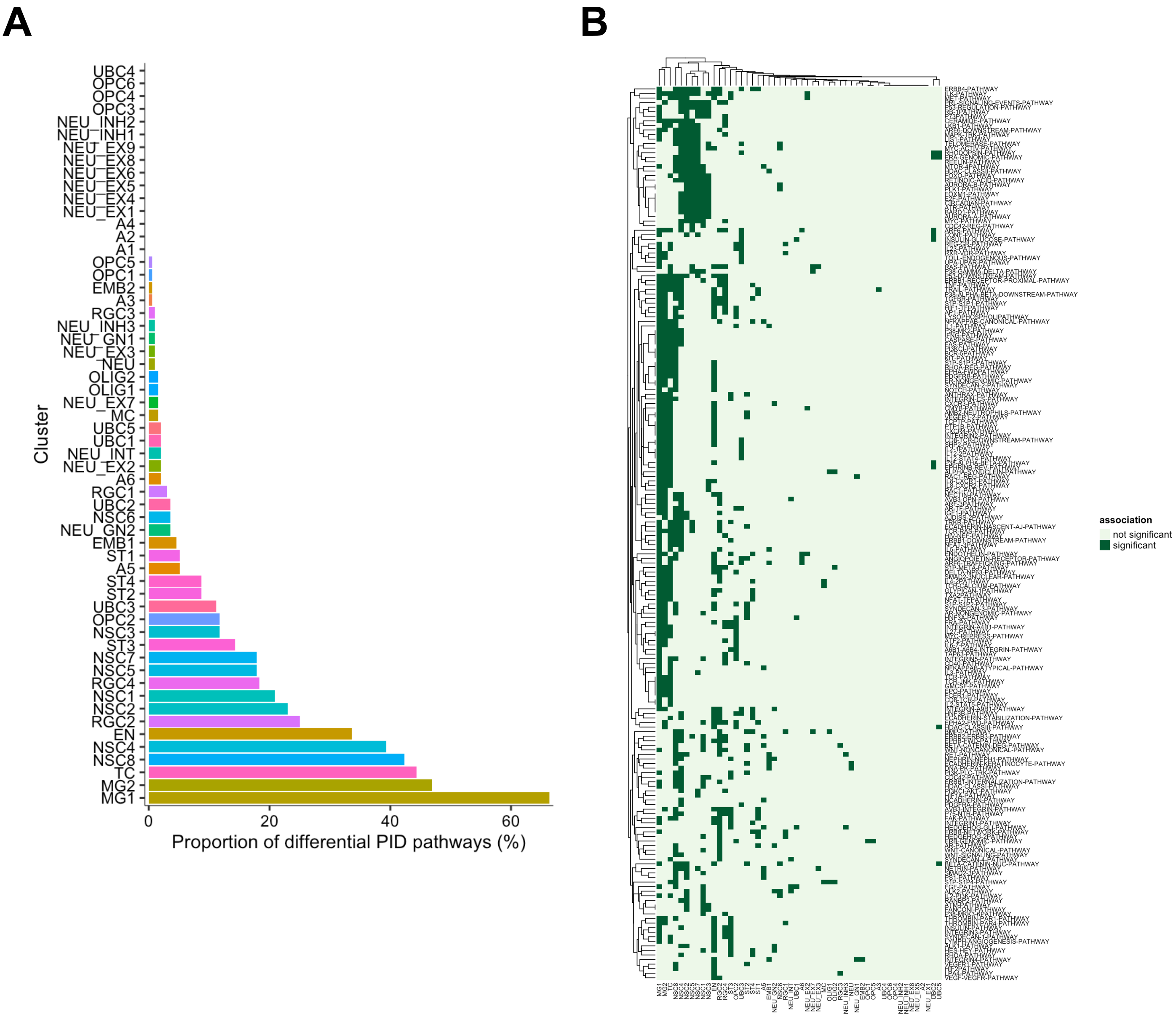

Supplementary Figure 8. Pathways associated with cell types. A) Proportion (out of 196 Pathway Interaction Database (PID) pathways tested) of pathways specific to cell types compared to all other nuclei. **B)** Hierarchical clustering of all 196 PID pathways tested for each cell type. Dark green indicates pathways relatively specific/important to the cell type by statistical significance threshold of adjusted P-value < 0.1

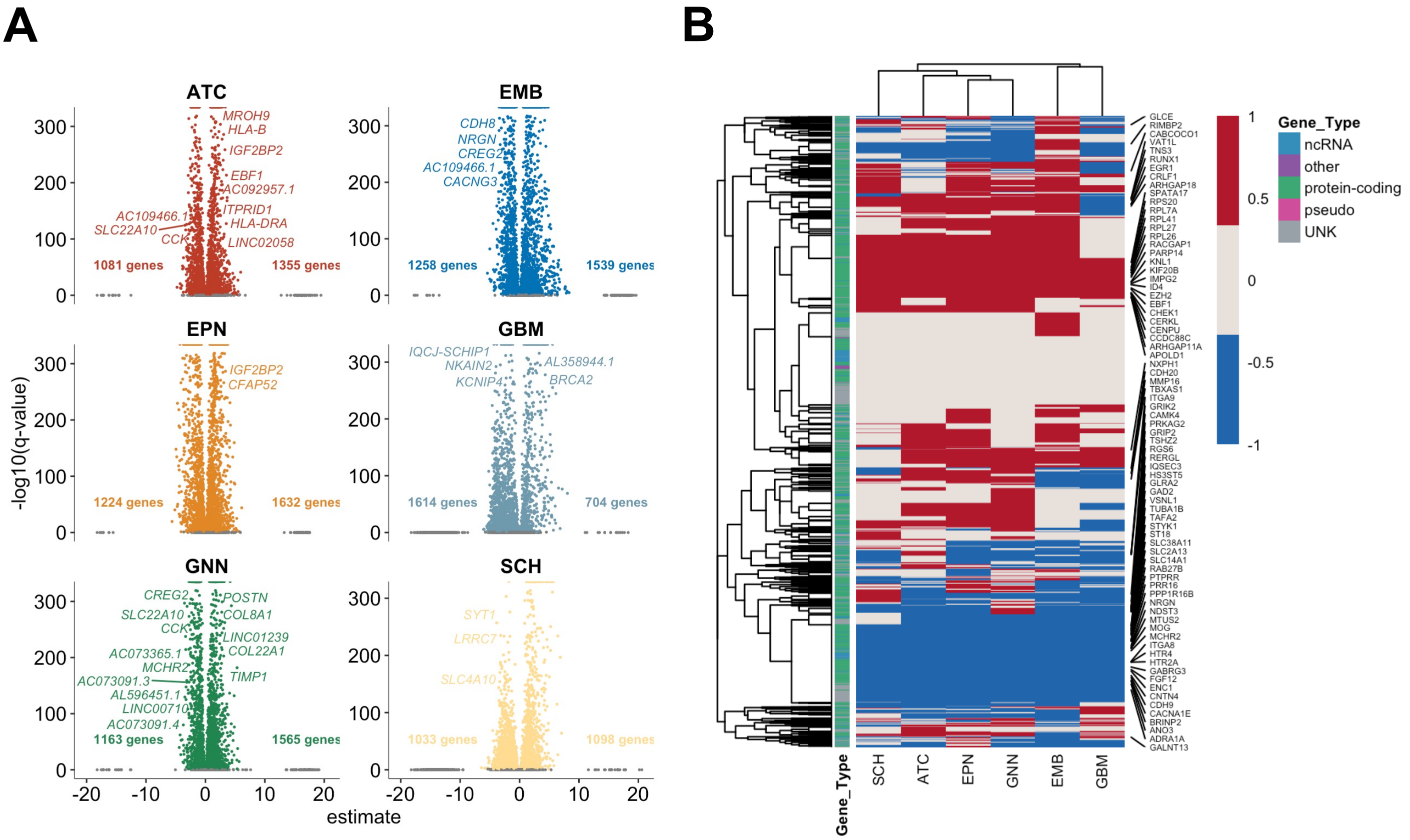

Supplementary Figure 9. Differential gene expression in the models unadjusted for cell type. A) Volcano plot of differentially expressed genes for each tumor type compared to non-tumor tissue, *not* adjusted for cell type. Number of genes on the left of the volcano plot indicate genes that are downregulated compared to non-tumor tissue. Number of genes on the right of the plot indicate genes that are upregulated compared to non-tumor tissue. **B)** Heatmap of differential expression direction and significance in all 4000 genes tested in the cell type unadjusted differential expression analyses. Red indicates significantly upregulated in the tumor type compared to non-tumor tissue. Blue indicates significantly downregulated in the tumor type compared to non-tumor tissue. Gray indicates the gene is not significantly differentially expressed. Tracking bar indicate the gene type.

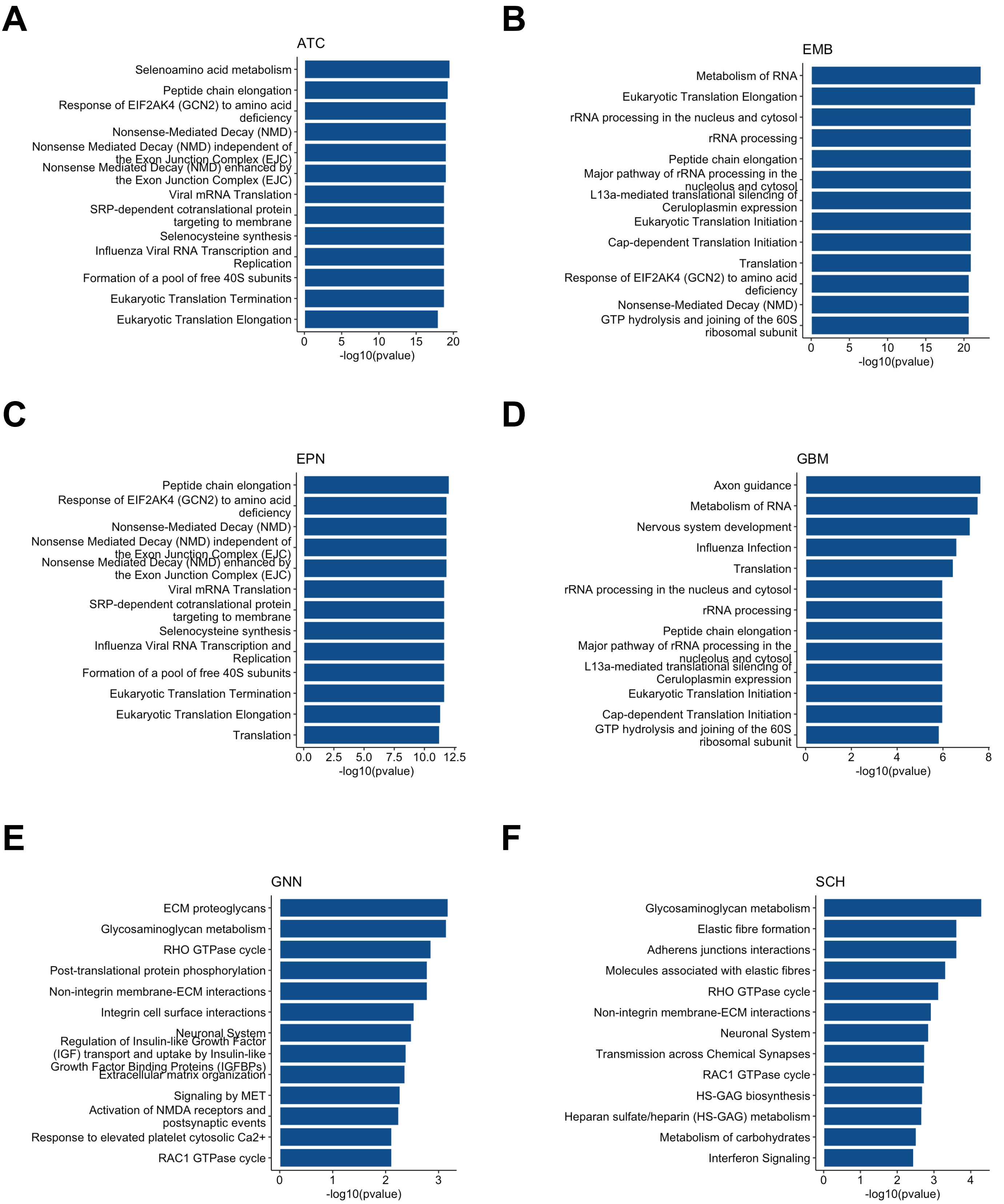

Supplementary Figure 10. **Pathways associated with differentially expressed genes**. Top 10 Reactome pathways associated with differentially expressed genes in **A)** astrocytoma, **B)** embryonal tumors, **C)** ependymoma, **D)** glioblastoma, **E)** glioneuronal/neuronal tumors, and **F)** Schwannoma.

Supplementary Table 1. Extended sample information

Sample Name	Sex	Age	Location	Location Class	2021_TumorType	2021_Grade	2021_Diagnosis	Multi-seq Pool	# of Nuclei
DHMC01	M	5	Posterior Fossa	Subtentorial	Ependymoma	3	Posterior fossa ependymoma, NOS, CNS WHO grade 3	Pool18, Pool19	448
DHMC02	M	7	Posterior Fossa	Subtentorial	Astrocytoma	1	Pilocytic astrocytoma, CNS WHO grade 1	Pool18, Pool19	2819
DHMC03	M	0.75	Temporal Lobe	Supratentorial	Astrocytoma	1	Pilocytic astrocytoma, CNS WHO grade 1	Pool18, Pool19	476
DHMC04	F	15	Parietal Lobe	Supratentorial	Ependymoma	3	Supratentorial ependymoma, NOS, CNS WHO grade 3	Pool20, Pool21, Pool22	234
DHMC05	M	3	4th Ventricle	Subtentorial	Ependymoma	3	Posterior fossa ependymoma, NOS, CNS WHO grade 3	Pool20, Pool21, Pool22	1026
DHMC06	F	12	Posterior Fossa	Subtentorial	Ependymoma	3	Posterior fossa ependymoma, NOS, CNS WHO grade 3	Pool20, Pool21, Pool22	1761
DHMC07	M	3	Left Temporal Lobe	Supratentorial	Glioneuronal/Neuronal	1	Dysembryoplastic neuroepithelial tumor, CNS WHO grade 1	Pool20, Pool21, Pool22	886
DHMC08	M	11	Vestibular	Subtentorial	Schwannoma	1	Schwannoma, CNS WHO grade 1	Pool20, Pool21, Pool22	2501
DHMC09	M	15	Occipital Lobe	Supratentorial	Glioneuronal/Neuronal	1	Ganglioglioma, CNS WHO grade 1	Pool20, Pool21, Pool22	830
DHMC11	M	18	Frontal Lobe	Supratentorial	Glioblastoma	4	Pediatric-type diffuse high grade glioma, NOS, CNS WHO grade 4	NA	5795
DHMC12	F	1	Posterior Fossa	Subtentorial	Astrocytoma	1	Pilocytic astrocytoma, CNS WHO grade 1	Pool3, Pool4	482
DHMC13	F	16	Occipital Lobe	Supratentorial	Ependymoma	2	Supratentorial ependymoma, NOS, CNS WHO grade 2	Pool3, Pool4	543
DHMC14	M	14	Posterior Fossa	Subtentorial	Embryonal	4	Medulloblastoma, classic, CNS WHO grade 4	Pool3, Pool4	380
DHMC15	M	16	Occipital Lobe	Supratentorial	Glioneuronal/Neuronal	NEC	Desmoplastic ganglioglioma, NEC	Pool3, Pool4	558
DHMC16	F	9	Temporal Lobe	Supratentorial	Glioneuronal/Neuronal	3	Anaplastic ganglioglioma, NOS, CNS WHO grade 3	Pool5, Pool6	3514
DHMC17	M	4	Frontal Lobe	Supratentorial	Glioneuronal/Neuronal	1	Desmoplastic infantile ganglioglioma, CNS WHO grade 1	Pool5, Pool6	3865
DHMC18	M	9	Posterior Fossa	Subtentorial	Ependymoma	NOS	Posterior fossa ependymoma, NOS	Pool5, Pool6	3096
DHMC19	M	3	Temporal Lobe	Supratentorial	Glioneuronal/Neuronal	1	Dysembryoplastic neuroepithelial tumor, CNS WHO grade 1	NA	3610
DHMC20	F	1	Posterior Fossa	Subtentorial	Embryonal	4	Embryonal tumor with multilayered rosettes, NOS, CNS WHO grade 4	Pool9	410
DHMC21	F	16	4th Ventricle	Subtentorial	Ependymoma	1	Subependymoma, CNS WHO grade 1	Pool9	273
DHMC22	M	5	Suprasellar	Supratentorial	Astrocytoma	1	Pilocytic astrocytoma, CNS WHO grade 1	Pool9	851
DHMC23	F	13	Posterior Fossa	Subtentorial	Astrocytoma	1	Pilocytic astrocytoma, CNS WHO grade 1	Pool9	655
DHMC24	M	8	4th Ventricle	Subtentorial	Ependymoma	2	Posterior fossa ependymoma, NOS, CNS WHO grade 2	Pool10, Pool11	2001
DHMC25	M	7	4th Ventricle	Subtentorial	Embryonal	4	Medulloblastoma, classic, CNS WHO grade 4	Pool10, Pool11	3870
DHMC26	M	10	Posterior Fossa	Subtentorial	Astrocytoma	1	Pilocytic astrocytoma, CNS WHO grade 1	Pool10, Pool11	742
DHMC27	M	15	Posterior Fossa	Subtentorial	Embryonal	4	Medulloblastoma, desmoplastic/nodular, CNS WHO grade 4	Pool10, Pool11	2356
DHMC28	M	18	Lateral Ventricle	Supratentorial	Glioneuronal/Neuronal	1	Dysembryoplastic neuroepithelial tumor, CNS WHO grade 1	Pool12	1662
DHMC29	F	13	Spinal cord	Subtentorial	Ependymoma	2	Myxopapillary ependymoma, CNS WHO grade 2	Pool12	4963
DHMC30	M	7	Posterior Fossa	Subtentorial	Ependymoma	3	Posterior fossa ependymoma, NOS, CNS WHO grade 3	NA	2199
DHMC31	F	1	Posterior Fossa	Subtentorial	Astrocytoma	1	Pilocytic astrocytoma, CNS WHO grade 1	Pool15	290
DHMC32	M	16	Posterior Fossa	Subtentorial	Glioneuronal/Neuronal	1	Gangliocytoma, CNS WHO grade 1	NA	1091
DHMC33	F	6	Parieto-Temporal Lobe	Supratentorial	Embryonal	4	Embryonal tumor, NOS, CNS WHO grade 4	Pool1, Pool2	5167
DHMC34	F	7	Posterior Fossa	Subtentorial	Ependymoma	2	Posterior fossa ependymoma, NOS, CNS WHO grade 2	Pool1, Pool2	5743
DHMC35	M	7	Posterior Fossa	Subtentorial	Astrocytoma	1	Pilocytic astrocytoma, CNS WHO grade 1	Pool1, Pool2	1399
DHMC36	F	12	Posterior Fossa	Subtentorial	Embryonal	4	Medulloblastoma, classic, CNS WHO grade 4	Pool1, Pool2	753
Normal	B	NA	NA	Supratentorial	Non-Tumor		Non-Tumor		

Supplementary Table 2. Summary statistics of stemness scores for each cell type

Cell Type	Minimum	1st quartile	Median	Mean	3rd quartile	Maximum
AST	-0.23	-0.01	0.04	0.05	0.10	0.47
EMB	-0.13	0.07	0.11	0.11	0.15	0.42
EN	-0.14	0.09	0.15	0.16	0.22	0.52
MAC/MG	-0.19	0.05	0.11	0.11	0.17	0.66
NEU	-0.24	-0.05	0.02	0.04	0.12	0.46
NSC	-0.22	0.19	0.28	0.28	0.37	0.77
OLIG	-0.20	0.02	0.08	0.08	0.13	0.55
OPC	-0.20	0.02	0.07	0.08	0.13	0.51
RGC	-0.21	0.05	0.11	0.12	0.19	0.59
ST	-0.17	0.04	0.09	0.09	0.14	0.48
TC	-0.11	0.04	0.10	0.10	0.16	0.42
UBC	-0.14	0.24	0.32	0.31	0.39	0.67

Supplementary Table 3. MULTI-seq oligonucleotide sequences.

Name	Read	Pattern	Sequence	Feature_type
MULTI_1	R2	5P(BC)	GGAGAAGA	Antibody Capture
MULTI_2	R2	5P(BC)	CCACAATG	Antibody Capture
MULTI_3	R2	5P(BC)	TGAGACCT	Antibody Capture
MULTI_4	R2	5P(BC)	GCACACGC	Antibody Capture
MULTI_5	R2	5P(BC)	AGAGAGAG	Antibody Capture
MULTI_6	R2	5P(BC)	TCACAGCA	Antibody Capture
MULTI_7	R2	5P(BC)	GAAAAGGG	Antibody Capture
MULTI_8	R2	5P(BC)	CGAGATTC	Antibody Capture
MULTI_9	R2	5P(BC)	GTAGCACT	Antibody Capture
MULTI_10	R2	5P(BC)	CGACCAGC	Antibody Capture
MULTI_11	R2	5P(BC)	TTAGCCAG	Antibody Capture
MULTI_12	R2	5P(BC)	GGACCCCA	Antibody Capture
