# Supplementary material for: Identifying tumor type and cell type-specific gene expression alterations in pediatric central nervous system tumors
Source: Nat Commun. 2024 Apr 30;15:3634. doi: 10.1038/s41467-024-47712-8 (PMC11061189; doi:10.1038/s41467-024-47712-8)
Supplement: Supplementary file 3 — Description of Additional Supplementary Files [file 41467_2024_47712_MOESM3_ESM.pdf]

Description of Additional Supplementary files

Supplementary Data 1. Genetic variants for each sample.

Supplementary Data 2. Enriched pathways per cell type. Pathways in each cell type were considered to be enriched from Wilcoxon rank sum tests.

Supplementary Data 3. Differential expression analysis results from cell type-adjusted model and cell type-unadjusted model. Differential expression analyses were conducted using quasi-poisson regression models.

Supplementary Data 4. Pathways associated with differentially expressed genes in astrocytomas in the cell type adjusted model

Supplementary Data 5. Pathways associated with differentially expressed genes in embryonal tumors in the cell type adjusted model

Supplementary Data 6. Pathways associated with differentially expressed genes in ependymomas in the cell type adjusted model

Supplementary Data 7. Pathways associated with differentially expressed genes in glioneuronal/neuronal tumors in the cell type adjusted model

Supplementary Data 8. Pathways associated with differentially expressed genes in glioblastoma in the cell type adjusted model

Supplementary Data 9. Pathways associated with differentially expressed genes in schwannomas in the cell type adjusted model
