# Supplementary material for: Identifying tumor type and cell type-specific gene expression alterations in pediatric central nervous system tumors
Source: Nat Commun. 2024 Apr 30;15:3634. doi: 10.1038/s41467-024-47712-8 (PMC11061189; doi:10.1038/s41467-024-47712-8)
Supplement: Supplementary file 5 — Reporting Summary [file 41467_2024_47712_MOESM5_ESM.pdf]

nature portfolio

Corresponding author(s): Min Kyung Lee

Last updated by author(s): 02/11/2024

Reporting Summary

Nature Portfolio wishes to improve the reproducibility of the work that we publish. This form provides structure for consistency and transparency in reporting. For further information on Nature Portfolio policies, see our Editorial Policies and the Editorial Policy Checklist.

Statistics

For all statistical analyses, confirm that the following items are present in the figure legend, table legend, main text, or Methods section.

n/a	Confirmed	
☐	☒	The exact sample size (*n*) for each experimental group/condition, given as a discrete number and unit of measurement
☐	☒	A statement on whether measurements were taken from distinct samples or whether the same sample was measured repeatedly
☐	☒	The statistical test(s) used AND whether they are one- or two-sided<br>*Only common tests should be described solely by name; describe more complex techniques in the Methods section.*
☐	☒	A description of all covariates tested
☐	☒	A description of any assumptions or corrections, such as tests of normality and adjustment for multiple comparisons
☐	☒	A full description of the statistical parameters including central tendency (e.g. means) or other basic estimates (e.g. regression coefficient) AND variation (e.g. standard deviation) or associated estimates of uncertainty (e.g. confidence intervals)
☐	☒	For null hypothesis testing, the test statistic (e.g. *F*, *t*, *r*) with confidence intervals, effect sizes, degrees of freedom and *P* value noted<br>*Give P values as exact values whenever suitable.*
☒	☐	For Bayesian analysis, information on the choice of priors and Markov chain Monte Carlo settings
☐	☒	For hierarchical and complex designs, identification of the appropriate level for tests and full reporting of outcomes
☐	☒	Estimates of effect sizes (e.g. Cohen's *d*, Pearson's *r*), indicating how they were calculated

Our web collection on statistics for biologists contains articles on many of the points above.

Software and code

Policy information about availability of computer code

Data collection	No software or code was used to collect data.
Data analysis	Code used for analysis is available at https://github.com/sarahmklee/IntegrativePCNS and https://github.com/AlexsLemonade/alsf-scpca/tree/main/workflows/genetic-demux. The following software were used for data analysis:<br>- 10X Cell Ranger<br>- STAR (v2.7.7a); STARsolo; bcftools; cellsnp-lite; FastQC (v0.11.8); cutadapt (v2.4); picard (v2.18.29); HTseq (v0.11.2); Picard Tools; Genome Analysis Toolkit<br>- R packages: Seurat (v4); minfi; conumee; VAM; monocle3 (v1.0.0); AnnotationDbi; ReactomePA

For manuscripts utilizing custom algorithms or software that are central to the research but not yet described in published literature, software must be made available to editors and reviewers. We strongly encourage code deposition in a community repository (e.g. GitHub). See the Nature Portfolio guidelines for submitting code & software for further information.

Data

Policy information about availability of data

All manuscripts must include a data availability statement. This statement should provide the following information, where applicable:

- Accession codes, unique identifiers, or web links for publicly available datasets
- A description of any restrictions on data availability
- For clinical datasets or third party data, please ensure that the statement adheres to our policy

The raw single nuclei-RNA seq data and the processed data for single nuclei-RNA seq generated in this study are available in the Gene Expression Omnibus under accession code GSE211362 [https://www.ncbi.nlm.nih.gov/geo/query/acc.cgi?acc=GSE211362]. The raw hydroxymethylation/methylation data generated in this study have been deposited in the Gene Expression Omnibus under accession code GSE152561 [https://www.ncbi.nlm.nih.gov/geo/query/acc.cgi?acc=GSE152561]. The raw bulk RNA-seq data generated in this study have been deposited in the Gene Expression Omnibus under accession code GSE241396 [https://www.ncbi.nlm.nih.gov/geo/query/acc.cgi?acc=GSE241396]. The processed single nuclei RNA-seq data is also available through the Pediatric Single Cell Atlas provided by the Alex's Lemonade Stand Foundation (https://scpca.alexslemonade.org). Detailed annotation of which pool each sample was multiplexed in can be found in Supplementary Table 1. All other data generated in this study are provided in the Supplementary Information/Source Data file. The source data are available in Figshare (https://figshare.com/projects/Identifying_tumor_type_and_cell_type-specific_gene_expression_alterations_in_pediatric_central_nervous_system_tumors/195539).

Human research participants

Policy information about studies involving human research participants and Sex and Gender in Research.

Reporting on sex and gender	Sex of each subject were collected from the clinical information during the patient's diagnosis and surgery. While sex-specific traits were not investigated, it was included to be adjusted for in the regression models.
Population characteristics	The study cohort were under 18 years of age as they needed to have CNS tumors during childhood. There were 8 astrocytoma, 6 embryonal tumors, 11 ependymoma, and 8 glioneuronal/neuronal tumors, 1 glioblastoma, and 1 schwannoma along with 3 non-tumor samples were included in the study. Non-tumor subjects were composed of 67% female and 33% male. The subjects with tumors were comprised of 37% female and 63% male subjects.
Recruitment	The subjects were recruited retrospectively based on the availability of pediatric CNS tumors that were available as frozen tissue at Dartmouth Hitchcock Medical Center.
Ethics oversight	This study complies with all Dartmouth Hitchcock Medical Center Institutional Review Board regulations. This study was approved by the Dartmouth Hitchcock Medical Center Institutional Review Board Study #00030211. All subjects provided consent for the use of tissues for research purposes.

Note that full information on the approval of the study protocol must also be provided in the manuscript.

Field-specific reporting

Please select the one below that is the best fit for your research. If you are not sure, read the appropriate sections before making your selection.

☒ Life sciences ☐ Behavioural & social sciences ☐ Ecological, evolutionary & environmental sciences

For a reference copy of the document with all sections, see nature.com/documents/nr-reporting-summary-flat.pdf

Life sciences study design

All studies must disclose on these points even when the disclosure is negative.

Sample size	Sample size was determined based on the availability of frozen pediatric CNS tumors available at Dartmouth Hitchcock Medical Center.
Data exclusions	Single nuclei RNA-seq with poor sequencing and poor nuclei quality were removed from analysis.
Replication	Due to the extremely small size of the frozen human tissue available, only one replicate per sample were obtained.
Randomization	The subjects could not be randomized as the samples were collected retrospectively and not at initial diagnosis.
Blinding	We could not apply blinding as we needed to confirm the diagnosis of the subjects in order to include the subjects in the study as having pediatric CNS tumors or being a pediatric control for which brain tissue were available.

Reporting for specific materials, systems and methods

We require information from authors about some types of materials, experimental systems and methods used in many studies. Here, indicate whether each material, system or method listed is relevant to your study. If you are not sure if a list item applies to your research, read the appropriate section before selecting a response.

Materials & experimental systems

n/a	Involved in the study
☒	☐ Antibodies
☒	☐ Eukaryotic cell lines
☒	☐ Palaeontology and archaeology
☒	☐ Animals and other organisms
☒	☐ Clinical data
☒	☐ Dual use research of concern

Methods

n/a	Involved in the study
☒	☐ ChIP-seq
☒	☐ Flow cytometry
☒	☐ MRI-based neuroimaging
